# Systemic-Pulmonary Collaterals in KCNT1-Related Disorders: Precise Nomenclature and Management

**DOI:** 10.1007/s00246-025-04131-w

**Published:** 2025-12-13

**Authors:** Jeffery Delaney, Utkarsh Kohli, Yuki Kawasaki, Casey Burg, Ponghatai Boonsimma, Azusa Ikeda, Tanitnun Paprad, David Bearden, Justin West, Sarah Drislane, Brad Bryan, Amanda Abuhl

**Affiliations:** 1 Department of Pediatrics, Division of Cardiology, University Nebraska Medical Center, Omaha, NE USA; 2 Division of Pediatric Cardiology, Department of Pediatrics, Nicklaus Children’s Hospital, Miami, FL USA; 3 Professor of Pediatrics, Florida International University Herbert Wertheim College of Medicine, Miami, FL USA; 4 Department of Pediatric Cardiology and Electrophysiology, Osaka General Hospital, Osaka City, Osaka Japan; 5 Department of Pediatrics, Division of Pulmonology, University Nebraska Medical Center, Omaha, NE USA; 6 Department of Pediatrics, Division of Medical Genetics and Metabolism, King Chulalongkorn Memorial Hospital, Bangkok, Thailand; 7 Department of Neurology, Kanagawa Children’s Medical Center, Yokohama, Kanagawa Japan; 8 Department of Pediatrics, Bangkok Hospital Phuket, Phuket, Phuket Province Thailand; 9 Department of Neurology, Division of Child Neurology, University of Rochester Medical Center, Rochester, NY USA; 10 KCNT1 Epilepsy Foundation, Scottsdale, AZ USA

**Keywords:** KCNT1, Systemic-pulmonary collaterals (SPCs), Vascular anomalies, Epileptic encephalopathy, Angiogenesis, Embolization

## Abstract

Pathogenic variants in KCNT1, a gene encoding a sodium-activated potassium channel, are classically associated with severe early-onset epileptic encephalopathies. Emerging evidence indicates that some individuals with KCNT1-related disorders also develop systemic-to-pulmonary vascular anomalies, often initially misidentified as major aortopulmonary collateral arteries (MAPCAs) due to radiographic similarities with congenital heart disease. Unlike true MAPCAs, these vessels arise in the absence of structural cardiac defects and appear to result from abnormal angiogenesis driven by dysregulated KCNT1-mediated signaling. Misclassification may lead to inappropriate interventions or management strategies. Here, a multidisciplinary expert working group reviewed published cases, institutional experience, and imaging findings to develop an expert opinion for nomenclature, diagnosis, and management of these vascular anomalies. We recommend adopting the term systemic-pulmonary collaterals (SPCs) to accurately describe these angiogenic vessels. Risk-based screening, judicious catheter-based embolization when physiologically indicated, and careful avoidance of standard pulmonary vasodilators are emphasized. This statement aims to improve clinical recognition, precision in terminology, and safe management of vascular complications in patients with KCNT1-related disorders.

## Introduction to KCNT1 and KCNT1-Related Disorders

*KCNT1* encodes a sodium-activated potassium channel (Slack or Slo2.2), which plays a critical role in regulating neuronal excitability. Pathogenic variants in *KCNT1* are associated with a spectrum of severe, early-onset epileptic encephalopathies, collectively referred to as KCNT1-related disorders [1–3]. These include Epilepsy of Infancy with Migrating Focal Seizures (EIMFS), Autosomal Dominant Nocturnal Frontal Lobe Epilepsy (ADNFLE), and other developmental and epileptic encephalopathies characterized by drug-resistant seizures, developmental delay, and often profound neurologic impairment.

Most pathogenic *KCNT1* variants are gain-of-function mutations, leading to increased channel activity and persistent neuronal hyperexcitability. Despite their primary neurologic presentation, emerging evidence suggests a broader multisystem phenotype in some patients [4]. Recent reports have highlighted vascular anomalies in individuals with *KCNT1* variants—findings that extend the disease spectrum beyond the central nervous system.

Given the severity and complexity of KCNT1-related disorders, early genetic diagnosis and multidisciplinary management are critical. A better understanding of the full phenotypic spectrum, including non-neurologic manifestations, is essential to improving outcomes and guiding targeted interventions.

### Pathogenic KCNT1 Sequence Variation and Systemic-Pulmonary Collaterals: Moving Beyond the MAPCA Paradigm

As our understanding of *KCNT1*-related disorders deepens, emerging evidence reveals a complex vascular phenotype associated with this gene, one that warrants careful distinction from vascular abnormalities associated with congenital heart disease (CHD) such as tetralogy of Fallot with pulmonary atresia and pulmonary atresia with ventricular septal defect (VSD). Specifically, a subset of patients with pathogenic *KCNT1* sequence variation present with vascular anomalies that supply excessive blood flow to the lungs. Several published case reports and small series have described pulmonary vascular anomalies in children with *KCNT1*-related epileptic encephalopathies, often initially interpreted as major aortopulmonary collateral arteries (MAPCAs) due to their radiographic resemblance to those seen in congenital heart disease.

A 10-month-old girl with KCNT1 (R474C) mutation–associated epileptic encephalopathy developed systemic-to-pulmonary artery collaterals, intermittent QTc prolongation, and left heart dilation that regressed spontaneously—heralding severe pulmonary hypertension—highlighting novel structural and electrical cardiac phenotypes associated with KCNT1 mutations [5]. A child was described with *KCNT1*-related epilepsy who developed extensive pulmonary vascular malformations and fatal pulmonary hemorrhage, illustrating the severe and potentially lethal nature of these anomalies when unrecognized or untreated [6]. Similarly, three infants were reported with *KCNT1* mutations and malignant migrating partial seizures of infancy who were found to have massive systemic-to-pulmonary collateral arteries [7]. Although initially labeled as MAPCAs, these vessels were present in the absence of congenital cardiac defects and coexisted with normal pulmonary arteries, raising questions about their true origin. A case of pulmonary hemorrhage was reported in a child with *KCNT1*-related developmental and epileptic encephalopathy, revealing multiple pulmonary vascular malformations without any underlying structural heart disease [8]. Most recently, life-threatening pulmonary hemorrhages have been described in *KCNT1* patients that have been managed with coil embolization [9, 10]. A summary of these case reports is presented in Table 1.

**Table 1:** Summary of cases with SPCs due to KCNT1 variant

Reference	Sex/Age At Diagnosis of SPCs	KCNT1 Mutation	Reason for Evaluation	Diagnostic Methods	Age at First Hemoptysis	Features/History
Kohli, et al. [5]	Female/2 months	c1420C > T; p. Arg474Cys, R474C	Heart Murmur	Contrast-enhanced CT	N/R	Prolonged QTc; pulmonary hypertension; left-heart enlargement; sudden death
Kawasaki et al. [7] Case 1	Female/2months	c.1129 T > A p.Cys377Ser *de novo*	Heart Murmur	Contrast-enhanced CT; echocardiogram	4 months	Died of hemoptysis at 6 months of age
Kawasaki et al. [7] Case 2	Female/3months	c.1420 C > T p.Arg474Cys *de novo*	Heart Murmur	Contrast-enhanced CT; echocardiogram	7 months	Died from hemoptysis at 9 months of age
Kawasaki et al. [7] Case 3	Male/1.5 years	c.1421 G > A p.Arg474Cys *de novo*	Abnormal chest CT	Contrast-enhanced CT; echocardiogram	1.7 years	Severe developmental & intellectual delay
Ikeda et al. [13] Patient 1	Male/11 years	c.1420 C > T p.Arg474Cys de novo	Hemoptysis	Angiography	8 years	Severe intellectual disability, general hypotonia, quadriplegia
Ikeda et al. [13] Patient 2	Female/8 years	c.808 C > G p.Gln270Glu de novo	Epistaxis	Angiography	8 years	Profound intellectual disability, truncal hypotonia, spastic quadriplegia
Cassibba et al. [8] Patient 1	Female/3 years	c.2677 G > A p.Glu893Lys de novo	Hemoptysis	Angiography	3 years	N/R
Okada et al. [10] Patient 1	Male/3 months	c.1130 G > C p.Cys377Ser de novo	Heart Murmur	Contrast-enhanced CT; echocardiogram	N/R	Profound developmental delay, bedridden, tube feeding
Garancini et al. [6] Patient 1	Male/6 years	N/R	Difficulty breathing; respiratory complications	Chest CT; echocardiogram	6 years	Died at the age of 7 following multiple episodes of hemoptysis

*SPC* Systemic Pulmonary Collaterals, *N/R* not available

In all cases, the vascular anomalies mimicked MAPCAs but appeared to arise independently of congenital malformations, suggesting a different underlying pathophysiology—likely angiogenic rather than developmental. This distinction is important as management strategies are quite different for these two entities. The incidence and prevalence of these KCNT1-related vascular anomalies are currently unknown, however the abundance of case studies presented in the literature and the frequency at which the KCNT1 Epilepsy Foundation is notified of these conditions within the KCNT1 community indicates that further understanding is necessary to inform clinicians on recommended treatments. Moreover, the molecular mechanism by which KCNT1 may regulate, either directly or indirectly, pulmonary vascular processes is currently unknown and in need of further research.

## Methods

This statement represents the expert opinion of a multidisciplinary working group composed of pediatric neurologists, cardiologists, interventional radiologists, pulmonologists, and clinical geneticists with experience managing patients with *KCNT1*-related disorders. The group convened to review emerging clinical evidence and collective institutional experience regarding systemic-to-pulmonary vascular anomalies in patients with *KCNT1* pathogenic variants.

The expert opinion process involved multiple structured virtual meetings and email correspondences held between September 2024 to July 2025, during which relevant case reports, imaging studies, and clinical data were reviewed. Key topics included the classification and terminology of vascular findings, distinctions from congenital heart disease-associated MAPCAs, appropriate screening strategies, and therapeutic considerations. Participants discussed and refined proposed clinical frameworks and recommendations through iterative feedback and written summaries circulated between meetings. This document reflects the collective expert opinion of the working group and is intended to guide clinicians in recognizing, evaluating, and managing vascular anomalies in *KCNT1*-related disorders. It does not replace formal clinical guidelines but provides a foundation for future studies and consensus development.

### Reframing the Vascular Phenotype: Angiogenesis, not Malformation

A multidisciplinary working group convened to address this issue and has reached an expert opinion to improve diagnostic clarity and management for pathogenic *KCNT1* sequence variation-associated vascular anomalies. Here we summarize key conclusions and opinions.

The systemic-to-pulmonary vascular connections observed in some pathogenic *KCNT1* sequence variation carriers are not true MAPCAs as defined in the context of complex CHD. Rather, these vessels arise from *abnormal angiogenesis*, likely influenced by dysregulated signaling pathways driven by the pathogenic *KCNT1* sequence variation and associated disease processes [5–10]. The morphology often involves diffuse, tortuous, and interconnected vessels that do not conform to embryologic patterns of pulmonary artery development. These are separate and distinct from the patient’s normal true pulmonary arteries and represent redundant additional blood flow (Table 2).

**Table 2:** Comparison between MAPCAs and SPCs

Characteristics	MAPCAs	SPCs
Origin of vessels	**Malformation**: Present in congenital heart defects tetralogy of Fallot with pulmonary atresia and pulmonary atresia with ventricular septal defect (VSD)	**Angiogenesis**: Present in the absence of congenital heart disease
Surveillance	CT angiography (CTA); Bubble Echocardiogram	CT angiography (CTA)
Treatment	Surgical management	Coil Embolization via catheterization

*MAPCA* major aortopulmonary collateral arteries, *SPC* system pulmonary collateral arteries

Using the term “MAPCAs” to describe these anomalies risks conflating them with surgically managed entities like vascular abnormalities associated with CHD, potentially leading to over-intervention or inappropriate medical therapies. Instead, we recommend the term systemic-pulmonary collaterals (SPCs) to more accurately reflect their acquired, angiogenic nature.

### Screening and Surveillance: A Tailored Approach

Given the heterogeneity of vascular findings in pathogenic *KCNT1* sequence variation carriers, a risk-based approach to screening is essential. We recommend:

- Diagnostic Imaging: CT angiography (CTA) remains the gold standard for anatomical assessment. Cardiac catheterization should be reserved for detailed hemodynamic evaluation or therapeutic planning.
- Bubble (contrast) echocardiography is primarily used to detect right-to-left shunts, such as intracardiac defects or pulmonary arteriovenous malformations, by visualizing microbubbles that bypass the pulmonary capillary filter. It is not indicated for identifying systemic–pulmonary arterial collaterals, which require angiographic imaging for diagnosis.
- Patient Selection: Screening should be considered in patients with increasing shortness of breath, airway bleeding (hemoptysis or pulmonary hemorrhage), cardiac dilation or dysfunction on screening echocardiogram, or recurrent infections. Neurologic comorbidities may also correlate with a higher angiogenic burden. Airway bleeding could be life threatening and may warrant urgent cardiac catheterization.

### Intervention: Strategic, not Reflexive

**Fig. 1:**
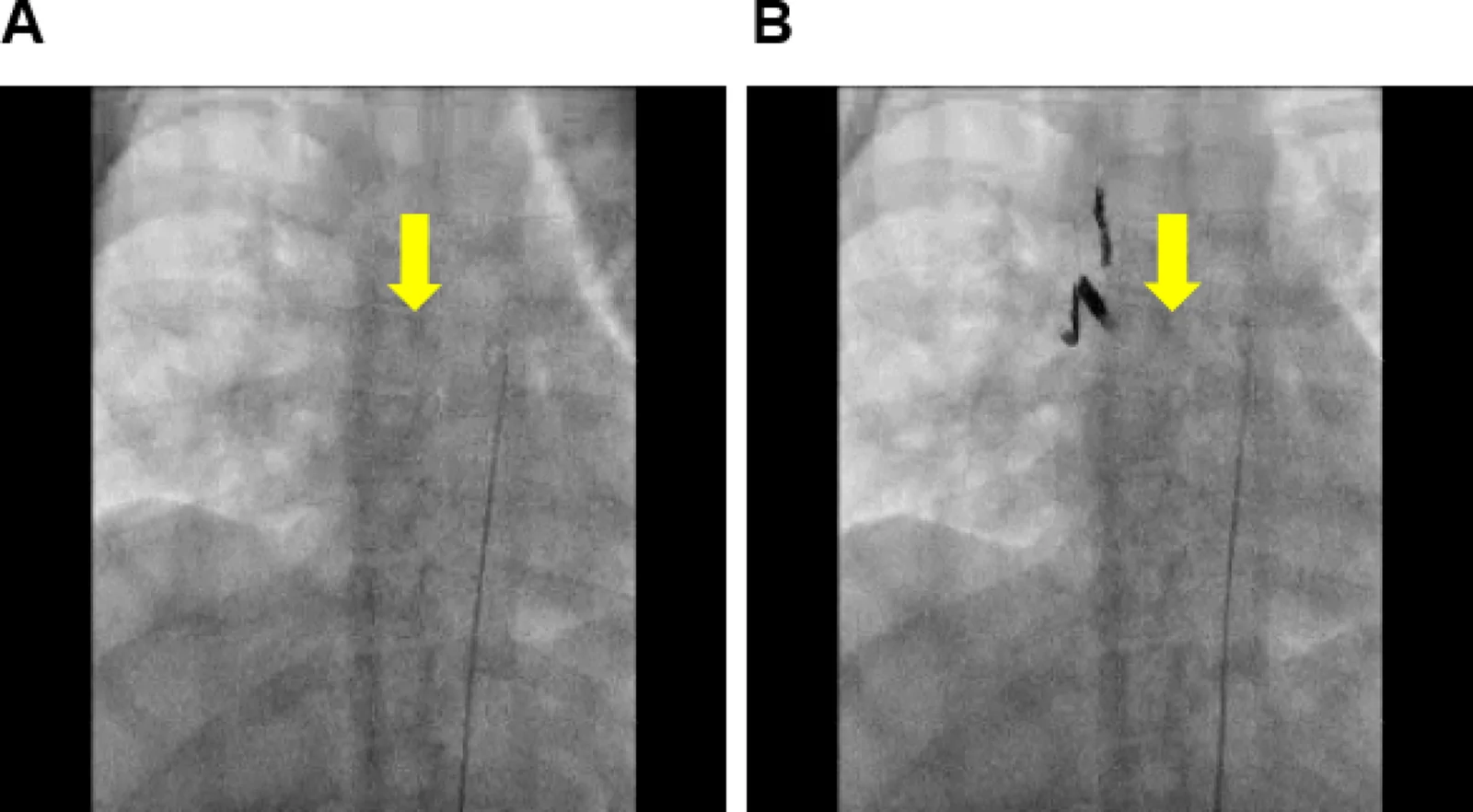
**A** Angiography of the descending aorta was performed, which demonstrated a prominent systemic–pulmonary collateral artery arising from the right intercostal artery (yellow arrow), supplying abnormal flow to the pulmonary circulation and contributing to pulmonary hemorrhage in this patient with a known KCNT1 pathogenic variant. **B** Selective catheterization of the collateral vessel was undertaken, and coil embolization was successfully performed at three distinct sites along the artery. Post-embolization angiography confirmed complete occlusion of the collateral vessel with no residual flow, effectively eliminating the abnormal systemic–pulmonary shunt

**Fig. 2:**
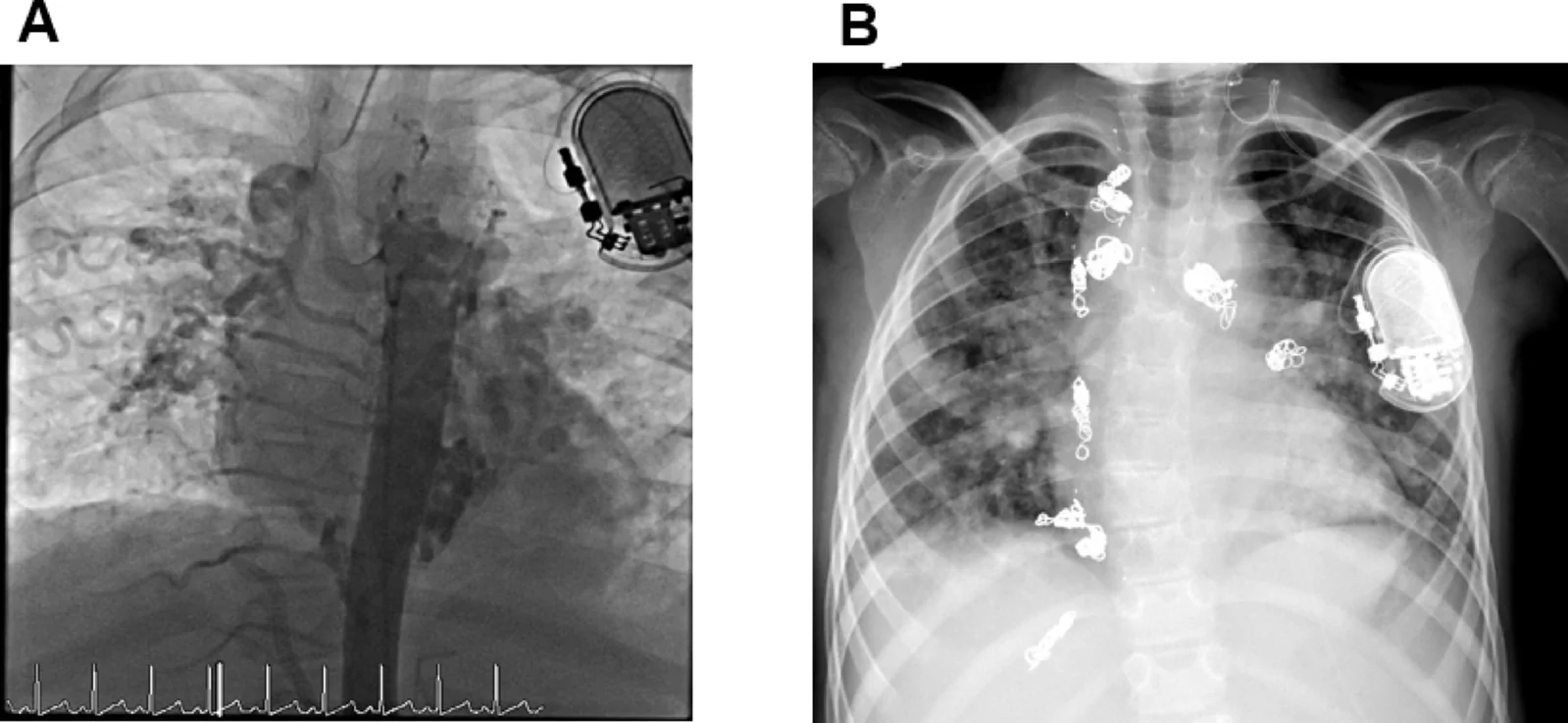
**A** Angiogram showing accessory pulmonary vessels prior to coil placement. **B** Chest radiograph post-catheterization, demonstrating vascular plugs throughout the aorta-pulmonary collateral network. At the time of the procedure, left ventricular end-diastolic dimension (LVEDD) was 4.6 cm (Z = 4.8), indicating significant volume loading. Two months post-embolization) LVEDD improved to 3.2 cm (Z = − 1), with stability on follow-up. While a single collateral can remain clinically significant, overall cardiac volume load was substantially reduced

In cases where SPCs contribute to significant physiologic burden—e.g, left heart volume overload, secondary hypoxemia due to pulmonary edema, and/or cardiac dysfunction —catheter-based embolization should be considered. Embolization of systemic–pulmonary collateral arteries has been successfully performed in clinical practice to control hemorrhage and reduce abnormal pulmonary blood flow (Figs. 1 and 2). Recurrent episodes of hemoptysis, even if not otherwise concerning, should trigger investigation. However, caution is warranted:

- Feasibility Assessment: Given the diffuse and interconnected nature of these vessels, procedural planning must consider both the technical challenges and the likelihood of symptomatic benefit.
- Avoid Over-Embolization: Lessons from other diffuse vascular malformation procedures, such as hereditary hemorrhagic telangiectasia (HHT), remind us that aggressive embolization may impact or damage normal tissue, or be associated with complications and high procedural radiation exposure. A measured approach is essential.
- Unlike vascular anomalies which are associated with CHD and are typically static, the angiogenic nature of SPCs has the potential to regress after intervention but may recur. Therefore, even after initial treatment, ongoing surveillance is necessary, and many necessitate further management.

### Medical Therapy: Precision and Prudence

Pathogenic *KCNT1* sequence variation carriers with SPCs do not benefit from standard pulmonary hypertension therapies. In fact, such agents may exacerbate the condition by increasing pulmonary blood flow and shunt volume. We recommend:

- Avoiding Vasodilators: Pulmonary vasodilators are contraindicated unless compelling evidence of pre-capillary pulmonary hypertension exists—and even then, extreme caution should be exercised.
- Supportive Care: Oxygen supplementation and mechanical ventilation should be individualized based on clinical needs. These interventions can mitigate hypoxemia and reduce pulmonary vasoconstriction but should not be viewed as definitive therapy.

## Conclusion: Precision in Terminology, Prudence in Management

As we evolve our clinical approach to management of patients with pathogenic KCNT1 sequence variation, we must resist the temptation to apply familiar labels like MAPCAs to unfamiliar phenotypes. Doing so obscures pathophysiologic mechanisms and risks harm through misguided interventions.

Instead, we urge the clinical community to adopt a more nuanced view—one that recognizes these vascular lesions as angiogenic complications of a neurogenetic disorder, not congenital malformations. Through tailored screening, judicious intervention, and cautious medical management, we can better serve this complex and vulnerable patient population.

While our understanding of these vascular manifestations continues to evolve, it is important to note that no systemic longitudinal studies have been conducted on SPCs in KCNT1 patients. All reported cases to date are limited to children, who often experience refractory seizures and may pass away from disease-related complications before reaching adulthood. Furthermore, longitudinal studies are essential to determine whether these vascular features persist, evolve, or resolve over time.

Pathogenic *KCNT1* variants primarily affect neurons, however a growing evidence of data from case reports [5–10] indicates this protein may also influence vascular biology. While the mechanism of action for this observation is unknown, one plausible mechanism is indirect neurovascular signaling via the cardiovascular brain circuit (CBC), where the nervous, immune, and cardiovascular systems form a mutually responsive and forward-acting tissue network to regulate acute and chronic cardiovascular function [11]. Alternatively, low-level *KCNT1* expression in non-neuronal tissues could directly affect abnormal vessel growth. Indeed, the Human Protein Atlas [12] reports low-level mRNA and protein expression across multiple non-neuronal tissues.

There is a growing body of evidence supporting a role for pathogenic KCNT1 variants in the development of SPCs. It must be emphasized that the opinions presented here are based on a limited number of reported cases, an inherent limitation in rare disease research. This carries a risk of overgeneralization and underscores the urgent need for systematic studies to determine the true incidence and prevalence of SPCs in KCNT1-related disorders. Continued data collection, collaborative registries, and longitudinal follow-up will be essential to refine clinical guidance, improve risk stratification, and optimize outcomes for this vulnerable patient population.

## Data Availability

No datasets were generated or analysed during the current study.
