# Correction: Systemic-Pulmonary Collaterals in KCNT1-Related Disorders: Precise Nomenclature and Management

**DOI:** 10.1007/s00246-026-04162-x

**Published:** 2026-01-17

**Authors:** Jeffery Delaney, Utkarsh Kohli, Yuki Kawasaki, Casey Burg, Ponghatai Boonsimma, Azusa Ikeda, Tanitnun Paprad, David Bearden, Justin West, Sarah Drislane, Brad Bryan, Amanda Abuhl

**Affiliations:** 1 Department of Pediatrics, Division of Cardiology, University Nebraska Medical Center, Omaha, NE USA; 2 Division of Pediatric Cardiology, Department of Pediatrics, Nicklaus Children’s Hospital, Miami, FL USA; 3 Professor of Pediatrics, Florida International University Herbert Wertheim College of Medicine, Miami, FL USA; 4 Department of Pediatric Cardiology and Electrophysiology, Osaka General Hospital, Osaka City, Osaka Japan; 5 Department of Pediatrics, Division of Pulmonology, University Nebraska Medical Center, Omaha, NE USA; 6 Department of Pediatrics, Division of Medical Genetics and Metabolism, King Chulalongkorn Memorial Hospital, Bangkok, Thailand; 7 Department of Neurology, Kanagawa Children’s Medical Center, Yokohama, Kanagawa Japan; 8 Department of Pediatrics, Bangkok Hospital Phuket, Phuket, Phuket Province Thailand; 9 Department of Neurology, Division of Child Neurology, University of Rochester Medical Center, Rochester, NY USA; 10 KCNT1 Epilepsy Foundation, Scottsdale, AZ USA

**Correction to: Pediatric Cardiology** 10.1007/s00246-025-04131-w

In this article, reference was missing and should have been “Ikeda A, Ueda H, Matsui K, Iai M, Goto T. Recurrent pulmonary hemorrhage in juvenile patients with KCNT1 mutation. Pediatr Int. 2021;63:352–354. 10.1111/ped.14427.”

The original article has been corrected.

